# Antibiotics Prescriptions Pattern among Patients Visiting Primary Health Care Centers (PHCC) before and during COVID-19 Pandemic: A Cross-Sectional Population-Based Study from Qatar

**DOI:** 10.3390/antibiotics12081228

**Published:** 2023-07-25

**Authors:** Salma Al-Nuaimi, Sara Alkuwari, Abdullah M. Al-Jubouri, Salma Hegazi, Lolwa Jolo, Hafsa Khalid, Saoud Bossa, Eisa Al-Shirawi, Merin Alex, Khalid H. Elawad, Habib Hasan Farooqui, Susu M. Zughaier

**Affiliations:** 1 Department of Basic Medical Sciences, College of Medicine, QU Health, Qatar University, Doha 2713, Qatar; 2 Health Protection, PHCC, Doha 26555, Qatar; 3 Department of Population Medicine, College of Medicine, QU Health, Qatar University, Doha 2713, Qatar; hfarooqui@qu.edu.qa

**Keywords:** antibiotic, prescription pattern, COVID-19, primary healthcare, Qatar

## Abstract

Background: The COVID-19 pandemic, caused by the novel coronavirus 2 (SARS-CoV-2), has been associated with an increased risk of secondary bacterial infections. Numerous studies have reported a surge in antibiotic usage during the COVID-19 pandemic. This study aims to examine the impact of the COVID-19 pandemic on the frequency and patterns of antibiotic prescriptions at Primary Health Care Centers (PHCC) in Qatar, comparing the period before and during the pandemic. Methods: This population-based, cross-sectional study analyzed all antibiotic prescriptions issued in two-month intervals before COVID-19 (November and December 2019) and during the initial wave (June and July 2020) of COVID-19. The study included 27 PHCCs in Qatar. Results: Prior to the COVID-19 outbreak, the PHCCs dispensed a total of 74,909 antibiotic prescriptions in November and December. During the first wave of COVID-19, the number decreased to 29,273 prescriptions in June and July 2020. Antibiotics were most commonly prescribed for adults and least commonly for the elderly, both before and during the COVID-19 period. In the pre-COVID-19 period, Betalactams and macrolides accounted for the majority (73%) of all antibiotic prescriptions across all age groups. However, during the COVID-19 period, Betalactams and other antibiotics such as Nitrofurantoin and Metronidazole (73%) were the most frequently prescribed. Conclusion: The rate of antibiotic prescriptions during the first wave of COVID-19 was lower compared to the two months preceding the pandemic at the PHCC in Qatar.

## 1. Introduction

Antimicrobial resistance (AMR) is a threat to human health with global implications and continues to increase due to the misuse of antibiotics that are frequently prescribed when they are not the best treatment option or are unnecessary [1]. An increase in AMR has been attributed to an increase in prescription rates, lack of antimicrobial stewardship, patient noncompliance, and antibiotic usage in the food chain, such as farming and agriculture, as well as in industry [2]. Various pathogenic bacteria have developed resistance to previously effective antibiotics rendering them worthless [3]. Globally, the economic cost of AMR is estimated to be 3 trillion US dollars in GDP loss [4].

For viral infections, although antibiotics are not the treatment of choice, it is a common practice to use antibiotics as prophylaxis to prevent secondary bacterial infections [5]. During the COVID-19 pandemic, antibiotics were used as a preventative intervention for high-risk patients, especially those admitted to the ICU and in need of mechanical ventilation. It has been reported that mechanical ventilation is associated with 86% of all hospital-acquired pneumonia, which requires antibiotic intervention [6]. The prophylactic use of antibiotics against opportunistic nosocomial bacterial infections is indicated in the clinical guidelines [6]. In the early stages of the COVID-19 outbreak, prophylactic use of medications such as azithromycin was commonly advised. Additionally, inadequate knowledge concerning COVID-19 treatment has resulted in the inappropriate use of antibiotics during the pandemic [7]. Therefore, it is expected that the misuse of antibiotics during that period may have significantly added to the growing burden of antimicrobial resistance (AMR) globally. 

In addition, during the COVID-19 pandemic, the public health interventions such as enforced lockdown measures, social distancing measures, and mask mandates have also resulted in a reduced burden of other community-acquired respiratory infections. It has been reported that the implementation of lockdown measures accompanied by infection prevention measures such as masking and social distancing led to a reduction in seasonal influenza transmission [8]. Cases of meningitis, pharyngitis, and otitis media among children have also decreased [9]. Many of these infections usually require antibiotic treatment. 

The State of Qatar has implemented various preventive measures since the beginning of COVID-19 pandemic to tackle the spread of the virus [10]. However, it is not clear whether these preventative measures affected the antibiotic prescription rate and incidence of community-acquired infections in Qatar [11]. The goal of this research is to determine the rate of antibiotics prescription before COVID-19, notably between November and December 2019, and at the first peak of cases around June and July 2020 in Qatar by analyzing patient data from 27 Primary Health Care Centers (PHCCs). The primary aim of this study is to determine whether there are any variations in antibiotic prescription rates among patients visiting the PHCCs before and during the COVID-19 pandemic in Qatar. The second aim is to determine the potential impact of COVID-19 on the types of antibiotics that were prescribed. 

## 2. Results

**Figure 1:**
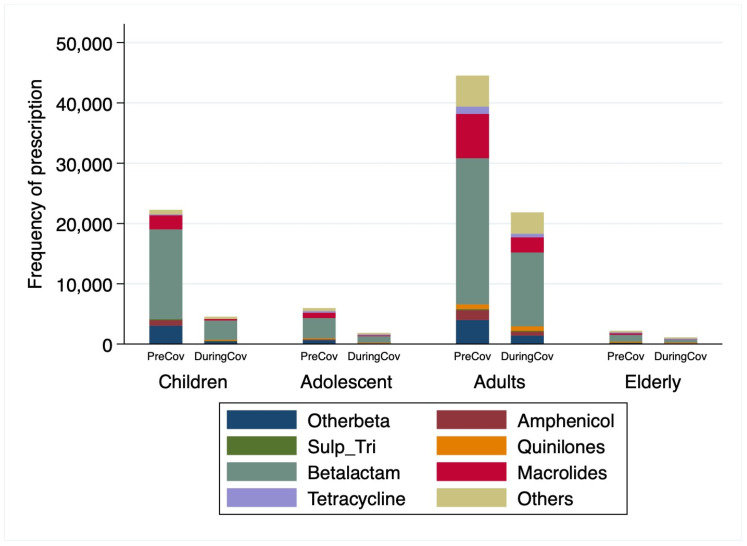
Antibiotic prescription pattern by age groups and antibiotic classes. PreCov: pre-COVID-19 period; DuringCov: During COVID-19 period; Sulp_Tri: Sulphamethaxazole/Trimethoprim. Otherbeta includes Cephalosporins, Monobactams and Carbapenem. Others includes Nitrofurantoin and Metronidazole.

Table 1 describes the characteristics of all the participants in the study. 74,909 antibiotic prescriptions were dispensed during the two months before the start of the COVID-19 (November and December 2019) and 29,273 during the first wave (June and July 2020) of COVID-19 period in the PHCCs in Qatar. The majority of the participants who were prescribed antibiotics were non-Qatari. The number of antibiotic prescriptions issued in the pre-COVID-19 period were highest for adults (44,506 prescriptions) and lowest for the elderly (2181 prescriptions), while during COVID-19, the antibiotic prescription rates were highest for adults (21,852 prescriptions) and lowest in the elderly (1065 prescriptions) as shown in Figure 1. Furthermore, there were 50.4% female participants in pre-COVID-19 period in comparison to 52.2% during COVID-19 period. Betalactams were the most prescribed antibiotics in both pre-COVID-19 period (58.3%) and during COVID-19 (58.6%), followed by Macrolides (14.8% vs. 10.2%) and Other antibiotics (8.8% vs. 14.6%) in pre-COVID-19 period and during COVID-19 period, respectively.

### Antibiotic Prescriptions across Clinical Diagnoses

Table 2 reports all the disorders that were diagnosed in all the PHCCs categorized into 22 categories according to the ICD-10. The five most prevalent medical conditions for which antibiotics were prescribed were those of the respiratory system, the digestive system, the ear and mastoid process, the skin and subcutaneous tissue, and the genitourinary system (Table 2).

Table 3 reports the effect of COVID-19 on the antibiotic prescription frequency and antimicrobial use among the common community-acquired communicable diseases that included conjunctivitis, otitis media, pharyngitis, and pneumonia. Since urinary tract infections (UTIs) are not a communicable disease unlike sexually transmitted diseases (STDs), it was selected for comparison. Among the selected common community acquired infectious diseases, in the pre-COVID-19, the majority of antibiotic prescriptions were issued for pharyngitis (36.21%), followed by otitis media (35.72%), and UTIs (15.06%). On the other hand, the majority of antibiotics dispensed during COVID-19 period were for UTIs (39.78%), otitis media (29.07%), and pharyngitis (16.97%).

**Figure 2:**
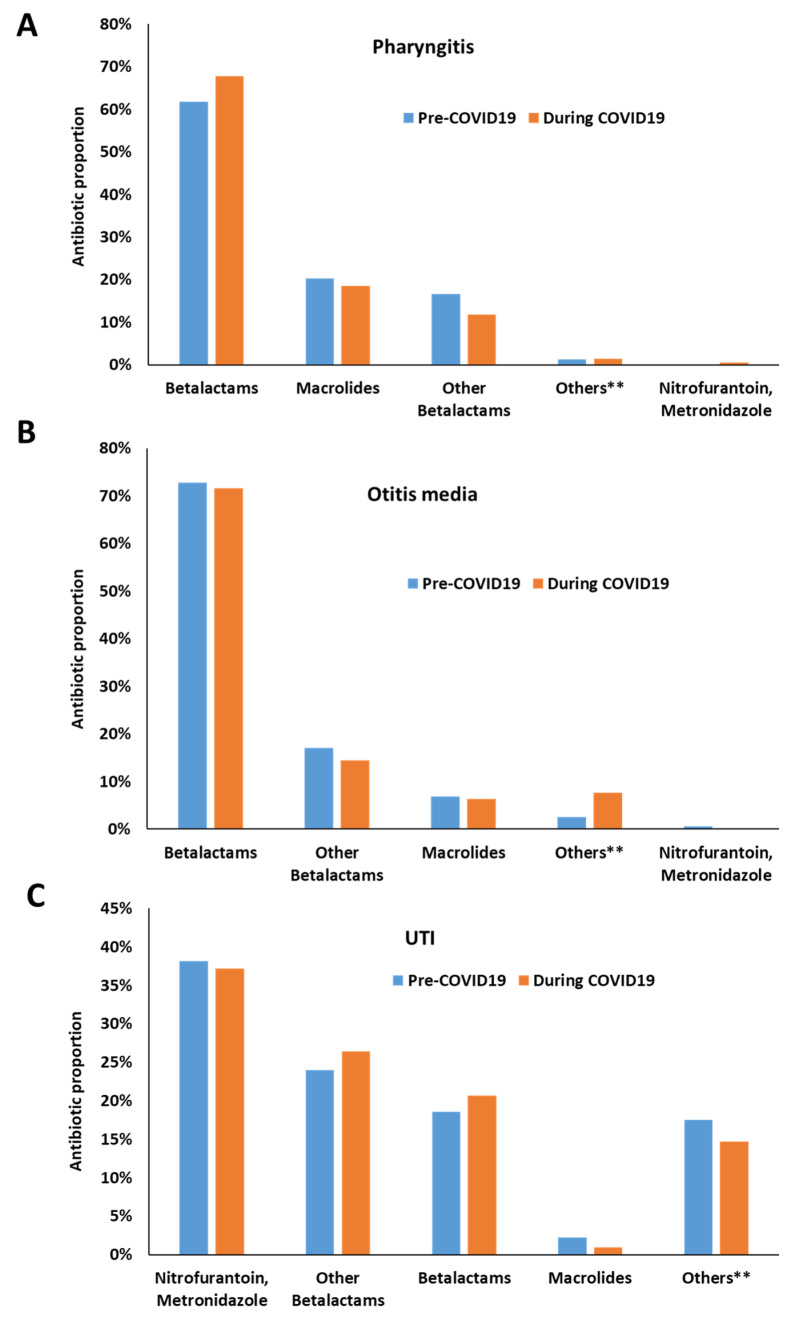
Antibiotic prescription pattern across infections pre-and during COVID-19. (**A**): Pharyngitis; (**B**): Otitis media; (**C**): UTI. Other Betalactams include Cephalosporins, Monobactams and Carbapenem. Others ** combine Quinolones + Tetracyclines + Amphinicol + Sulphamethaxasole/Trimethoprime. Note that the bars depict proportions of prescriptions within each period separately.

Figure 2 depicts the percentage of antibiotic prescriptions across communicable diseases according to their classification. In patients with pharyngitis, more Betalactams (61.8%) followed by Macrolides (20%) were dispensed during the pre-COVID-19 period. The trend during COVID-19 period was similar (67.8% vs. 18.50%). As for Otitis media, more Betalactams (72.80%) followed by Other Betalactams category that include Cephalosporins, Monobactams and Carbapenem (17%) were dispensed during the pre-COVID-19 period. The trend during COVID-19 period was similar (71.60% vs. 14.50%).

## 3. Discussion

This study observed a sharp decline in antibiotic prescription rates in Qatar’s PHCCs during COVID-19 period compared to the pre-COVID-19 period. A strict lockdown was enforced in Qatar during the first wave of COVID-19, resulting in limited physical access to primary healthcare’s, which may explain the reduction in infections and antibiotic prescription rates. During the first wave of COVID-19, many people refrained from going to healthcare centers [10] due to many reasons, including social distancing rules, flooding of healthcare centers for PCR testing and identification of diseased patients, and due to the fear of acquiring the SARS-CoV-2 virus. Additionally, most of the healthcare consultations during this period were delivered by phone (telemedicine), which possibly could have affected the number of antibiotic prescriptions. Whereas specific healthcare facilities such as temporary hospitals were made available to isolate and treat COVID-19 infections in Qatar. 

A key finding of this study is that Betalactams and Macrolides were the most common antibiotics prescribed in the pre-COVID-19 period across all age groups. Whereas during COVID-19 period, Betalactams remained to be the most prescribed, Macrolides were no longer prescribed as much as nitrofurantoin and metronidazole “Other antibiotics category as listed by ATC”, which were the second most common category of antibiotic prescriptions. Betalactams were the most prescribed antibiotics during the study period, which could be explained by several reasons. Firstly, many classes of Betalactams can be considered broad-spectrum and effective as the first-line therapy against various pathogens [12,13]. Moreover, many of the Betalactams were readily accessible and widely used in the PHCCs. There was a decline in Macrolide prescriptions during the COVID-19 period. This could be attributed to the publication of the Centre of Evidence-Based Medicine report in April 2020 about the fact that there is insufficient evidence to recommend the usage of Macrolides for COVID-19 and it could only be used to treat COVID-19 that was complicated by a bacterial superinfection [14].

In the pre-COVID-19 period, respiratory diseases were identified as the leading cause of antibiotic prescriptions. However, during the COVID-19 period, respiratory diseases no longer constituted the most common cause for such prescriptions. This change can be attributed to the altered healthcare-seeking behavior observed during the first wave of COVID-19. During the pre-COVID-19 period, individuals experiencing respiratory symptoms would typically seek treatment at Primary Health Care Centers (PHCCs). However, in the context of the COVID-19 pandemic, patients suspected of having respiratory symptoms were advised against visiting PHCCs directly. Instead, they were encouraged to contact specialized COVID-19 hotlines, which facilitated testing, treatment, and isolation for confirmed cases, without requiring access to PHCCs. As an illustrative example, the incidence of pharyngitis, a disease sharing common symptoms with COVID-19, exhibited a noticeable decrease during this period. This decline can be attributed to public health interventions that aimed to reduce the burden of airborne infections. Notably, the implementation of social distancing measures has been well-documented in reducing the transmission of infectious diseases, particularly those transmitted through the air [15]. Furthermore, the similarity in clinical presentation between pharyngitis and the primary symptoms of COVID-19, such as sore throat and fever, suggests that some patients with pharyngitis may have been treated as suspected COVID-19 cases and placed in isolation facilities until they tested negative for the viral infection.

Another important finding is an increase of the antibiotic prescription rates for diseases of the gastrointestinal system from about 19% in the pre-COVID-19 period to around 38% during COVID-19 period. This could be possibly explained by two reasons. The first being that due to the large decrease of antibiotic prescriptions for diseases of the respiratory system, a large proportional change is observed in the prescriptions for the diseases of gastrointestinal system, though the actual number of prescriptions were small. The second reason could be due to the fact that COVID-19 did in fact affect the gastrointestinal system in many patients who were infected with the virus. Recently, researchers were able to prove that a noticeable number of patients who had COVID-19 respiratory symptoms also suffered gastrointestinal symptoms, such as diarrhea. Researchers attributed this due to the abundance of Angiotensin Converting Enzyme 2 (ACE2) receptors on the gastrointestinal tract and how the SARS-CoV-2 virus uses the ACE2 receptors as the entry point into the body and affects multiple systems with this interaction [16]. 

Our study also documented the decrease in antibiotic prescriptions for communicable diseases after the implementation of social distancing and lockdown legislations. Other studies reported a decline in otolaryngeal cases, seasonal influenza, and other infectious diseases during the COVID-19 lockdown in many countries [8,9]. Additionally, the rates of antibiotic prescriptions for UTIs were also decreased although UTIs are non-airborne infections. This could be explained by the fact that during the first wave of COVID-19, patients were not willing to access healthcare facilities to get treatment for any symptoms that were not related to COVID-19 and would prefer to wait at home until the symptoms intensify. This was due to their fear of acquiring the virus when visiting the primary health centers during the first wave of COVID-19 [17]. This possibly meant that patients would wait for their symptoms to either self-eliminate or intensify. Consequently, emergency care would likely be sought instead of primary care.

A recent study from Italy, which compared the rates of antibiotic prescription before and during COVID-19 in a pediatric population, also reported a similar decline in the rates of antibiotic prescriptions [18]. It is also worth mentioning that the decline in antibiotic prescriptions was found in all diagnoses, with the exception of UTIs as the rates for antibiotic prescriptions did not change. This could be explained by the fact that antibiotics remain the first-line therapy for these complicated diseases and are even used as prophylaxis, thus, antibiotic prescription rates for UTIs do not get affected by other diseases. The study conducted in Italy has also shown a similar trend of decreased antibiotic prescription rates during the COVID-19 period compared to the pre-COVID-19 period due to the restrictions being imposed by health authorities and social distancing [19,20]. This consequently caused a decrease in the number of patients accessing the healthcare facilities during this period and taking the antibiotics that were prescribed for them; a finding that was similar to what happened in Qatar. 

Clinically, our data shed light on the types of antibiotics mostly prescribed for different diseases and different age groups at primary healthcare centers in Qatar. Our study reports the most common infectious diseases that required antibiotic prescriptions before and during the COVID-19 pandemic. Our study supports the value of antibiotics as a crucial type of treatment that should be preserved and only used when needed to reduce the burden of antimicrobial resistance [21,22]. 

Our study also sheds light on the frequency of antibiotic use in primary healthcare settings. Further research is warranted to investigate the rates of antibiotic prescriptions in secondary and tertiary healthcare settings such as ICU patients admitted consequent to severe COVID-19. This ICU cohort is at the highest risk of developing secondary bacterial infections due to intubation and immune suppression [23]. Bacterial superinfections in hospitals could be due to the usage of some medical devices, such as ventilators or Foley’s catheters, thereby requiring antibiotic treatment [6]. Since AMR is an urgent threat to human health, the public health authorities in Qatar have strict legislation kept in place to control the rates of antibiotic prescriptions that prevent people from buying antibiotics from hospitals or local pharmacies without prior consultation from a physician [24]. The antimicrobial stewardship program is established at all levels and spans from primary to tertiary health care facilities in Qatar. The main role of this program is reducing the burden of AMR by education, implementation of policies, surveillance of AMR pathogens and monitoring antibiotic misuse [25].

The strength of this study includes the large sample size of patients (104,110 records) with different age groups and socioeconomic statuses. The study cohort is representative of the diverse Qatari population. This is the first study done in Qatar about antibiotic prescriptions from the primary healthcare cohort of patients. Furthermore, the results can be generalized for different age groups concerning antibiotic prescriptions. Additionally, the rates, patterns and disease conditions for which antibiotics were prescribed provide different pieces of information setting a baseline for further research to explore further trends.

Our study has a few limitations. The study examined antibiotic utilization at the primary care level, but did not include data from patients who were treated in secondary and tertiary public hospitals, in addition to data from private hospitals and clinics. Another limitation is the limited data points in terms of time periods (2 months before and 2 months after COVID-19) under consideration for a prescription pattern analysis. Ideally, to conduct this study in a more accurate way, the design should have been a time series analysis. A weakness of this study is that the data we were provided for two different months before and during COVID-19 which did not account for seasonality of viral infections. To explain, the two months that were used before COVID-19 were November and December 2019, whereas, during COVID-19, the two months were June and July 2020. This could open room for a seasonality effect, potentially affecting the data in November and December as it is wintertime, and the flu is usually common. Although the researchers conducting this study asked for data to be analogous in months before and during the pandemic, the data provided could not satisfy this request.

## 4. Materials and Methods

### 4.1. Study Design

The study is retrospective, cross-sectional, and population-based. The study included data about all encounters (visits) where antibiotics were prescribed two months before COVID-19 (November and December 2019) and two months during the first wave (June and July 2020) of COVID-19 in Qatar. However, during data analysis, subjects with missing data or variables were excluded.

### 4.2. Participants

The study included de-identified data from the medical electronic system of the patients attending the primary health care centers. A total of 104,110 patients’ data was retrieved from electronic medical records through CERNER, capturing all encounters where antibiotics were prescribed from the 27 PHCCs during the study period described above. The patient population consisted of both nationals and expatriates of different ages and socioeconomic statuses who visited any of the 27 PHCCs in Qatar throughout the aforementioned period of the study. Age, gender, nationality, and other demographics were identified for all patients.

### 4.3. Data Collection 

The obtained data contained various diagnoses for which antibiotics were prescribed. The name of the antibiotic, route of administration and duration of treatment were all identified within the data. It is also worth mentioning that during the first wave of COVID-19, the initial treatment regimen that was used worldwide was a combination of Azithromycin (macrolide) and hydroxychloroquine [26]. 

### 4.4. Statistical Analysis

The International Statistical Classification of Diseases and related health problems, 10th revision (ICD10 classification; version 2019) was used to categorize the diagnosis according to antibiotic prescription. The aim was to group the diagnoses to the main levels of ICD10 codes and we had a total of 22 groups. In addition, the prescribed antibiotics were classified in relation to the diagnosis according to the 3rd level of Anatomic Therapeutic Chemical (ATC) classification as per the methodology proposed by the World Health Organization’s Collaborating Centre (WHOCC) of Drug Statistics Methodology (ATC index-2016). The medications prescribed were grouped into the following antibiotic subgroups (ATC codes): Tetracyclines (J01A), Amphenicols (J01B), Betalactams (J01C), other Betalactams (J01D), Sulfonamides & Trimethoprim (J01E), Macrolides (J01F), Quinolones (J01M) and Other antibiotics (J01X). 

We measured antibiotic prescriptions in terms of proportions across ICD10 disease categories and by ATC categories. We also reported antibiotic proportion, before and during COVID-19, by age groups and by community-acquired infectious conditions like pharyngitis and otitis media. Age groups were categorized into 4 groups (children, adolescents, adults, and elderly) according to the WHO classification. Stata 17.0 was used to perform statistical analysis. Pearson Chi square test was used to determine statistical significance and exact *p*-values are reported where necessary. 

## 5. Conclusions

The analysis of this study reveals that during the first wave of the COVID-19 pandemic, the rate of antibiotic prescriptions decreased compared to the two months preceding it. In the pre-COVID-19 period, Betalactams and Macrolides were the most commonly dispensed antibiotics across all age groups, whereas during the COVID-19 period, Betalactams and Other antibiotics (including Nitrofurantoin and Metronidazole) were more frequently prescribed. Furthermore, the study found that respiratory diseases accounted for a higher proportion of antibiotic prescriptions in the pre-COVID-19 period, whereas digestive system diseases were more prevalent during the COVID-19 period. These findings reflect the impact of the pandemic on both the number and types of antibiotics prescribed in primary healthcare settings. This research serves as a valuable background and baseline for future investigations into the patterns of antimicrobial prescriptions at Primary Health Care Centers (PHCCs) in Qatar. It also sheds light on the influence of COVID-19 pandemic lockdown measures on other community-acquired infections that typically require antibiotic treatment.

## Figures and Tables

**Table 1:** Characteristics of participants and medications prescribed.

Factor	Level	Pre COVID-19 Period	During COVID-19 Period	*p*-Value ^#^
N		74,909	29,273	
Nationality	Non-Qatari Qatari	51,413 (68.6%) 23,496 (31.4%)	21,721(74.2%) 7552 (25.8%)	<0.001
Age	Children Adolescents Adults Elderly	22,259 (29.7%) 5963 (8.0%) 44,506 (59.4%) 2181 (2.9%)	4522 (15.4%) 1834 (6.3%) 21,852(74.6%) 1065 (3.6%)	<0.001
Gender	Female Male	37,736 (50.4%) 37,172 (49.6%)	15,285(52.2%) 13,986(47.8%)	<0.001
Medication	Betalactams	43,650 (58.3%)	17,148 (58.6%)	<0.001
	Macrolides	11,089 (14.8%)	2992 (10.2%)	
	Other betalactams *	7958 (10.6%)	2064 (7.1%)	
	Nitrofurantoin and Metronidazole	6561 (8.8%)	4287 (14.6%)	
	Amphenicol	2979 (4.0%)	1012 (3.5%)	
	Tetracycline	1534 (2.0%)	724 (2.5%)	
	Quinolones	875 (1.2%)	917 (3.1%)	
	Sulfamethoxazole/ trimethoprim	263 (0.4%)	129 (0.4%)	

* Other Betalactams includes Cephalosporins, Monobactams and Carbapenem. ^#^ *p* value for categorical variables was calculated using Pearson Chi square test and for continuous variables was calculated using Wilcoxon rank-sum test.

**Table 2:** Matching of ICD-10 codes for the patients’ diseases.

ICD-10	Number of Cases		
	Pre COVID-19 (%)	During COVID-19 (%)	Total N (%)
Endocrine, nutritional and metabolic diseases	410 (0.55%)	259 (0.89%)	669 (0.64%)
Certain conditions originating in the perinatal period	6 (0.01%)	7 (0.02%)	13 (0.01%)
Certain infectious and parasitic diseases	1113 (1.49%)	326 (1.11%)	1439 (1.38%)
Codes for special purposes **	0 (0.00%)	1668 (5.70%)	1669 (1.60%)
Congenital malformations, deformations and chromosomal abnormalities	18 (0.02%)	1 (0.00%)	19 (0.02%)
Diseases of the blood and blood-forming organs and certain disorders involving the immune mechanism	40 (0.05%)	19 (0.06%)	59 (0.06%)
Diseases of the circulatory system	260 (0.35%)	181 (0.62%)	441 (0.42%)
Diseases of the digestive system	14,520 (19.39%)	11,182 (38.24%)	25,702 (24.69%)
Diseases of the ear and mastoid process	7366 (9.84%)	2070 (7.08%)	9436 (9.06%)
Diseases of the eye and adnexa	2870 (3.83%)	1117 (3.82%)	3987 (3.83%)
Diseases of the genitourinary system	3440 (4.59%)	2391 (8.18%)	5831 (5.60%)
Diseases of the musculoskeletal system and connective tissue	387 (0.52%)	220 (0.75%)	607 (0.58%)
Diseases of the nervous system	27 (0.04%)	17 (0.06%)	44 (0.04%)
Diseases of the respiratory system	33,486 (44.72%)	3198 (10.94%)	36,684 (35.24%)
Diseases of the skin and subcutaneous tissue	5128 (6.85%)	3063 (10.48%)	8191 (7.87%)
External causes of morbidity and mortality	176 (0.24%)	160 (0.55%)	336 (0.32%)
Factors influencing health status and contact with health services	851 (1.14%)	906 (3.10%)	1757 (1.69%)
Injury, poisoning and certain other consequences of external causes	1384 (1.85%)	833 (2.85%)	2217 (2.13%)
Mental and behavioural disorders	12 (0.02%)	7 (0.02%)	19 (0.02%)
Neoplasms	22 (0.03%)	17 (0.06%)	39 (0.04%)
Pregnancy, childbirth and the puerperium	44 (0.06%)	27 (0.09%)	71 (0.07%)
Symptoms, signs and abnormal clinical and laboratory findings, not elsewhere classified	3310 (4.42%)	1570 (5.37%)	4880 (4.69%)
Total	74,871 (100%)	29,239 (100%)	104,110 (100%)

** Codes for special purposes **: This category has only the diagnosis of COVID-19 disease.

**Table 3:** Antibiotic prescription frequencies and proportions across several communicable infections.

Diseases	Number of Cases		
	Pre COVID-19 (%)	During COVID-19 (%)	Total N (%)
Pharyngitis	5808 (36.21%)	661 (16.97%)	6469 (32.46%)
Otitis media	5728 (35.72%)	1132 (29.07%)	6860 (34.42%)
UTI *	2416 (15.06%)	1549 (39.78%)	3965 (19.89%)
Conjunctivitis	1704 (10.62%)	526 (13.51%)	2230 (11.19%)
Pneumonia	382 (2.38%)	26 (0.67%)	408 (2.05%)
Total	16,038 (100%)	3894 (100%)	19,932 (100%)

* UTI: was selected as reference infectious diseases as it is not transmitted through respiratory route.

## Data Availability

Data were obtained from PHCC (https://www.phcc.gov.qa/) on 29 March 2021 in Qatar under MTA agreement and therefore not available.
